# Computing Program Reliability Using Forward-Backward Precondition Analysis and Model Counting

**DOI:** 10.1007/978-3-030-45234-6_9

**Published:** 2020-03-13

**Authors:** Aleksandar S. Dimovski, Axel Legay

**Affiliations:** 8 University of Paderborn, Paderborn, Germany; 9 ICREA, Open University of Catalonia, Barcelona, Spain; 10 Mother Teresa University, 12 Udarna Brigada 2a, 1000 Skopje, North Macedonia; 11 Université catholique de Louvain, 1348 Ottignies-Louvain-la-Neuve, Belgium

## Abstract

The goal of probabilistic static analysis is to quantify the probability that a given program satisfies/violates a required property (assertion). In this work, we use a static analysis by abstract interpretation and model counting to construct probabilistic analysis of deterministic programs with uncertain input data, which can be used for estimating the probabilities of assertions (*program reliability*).

In particular, we automatically infer necessary preconditions in order a given assertion to be satisfied/violated at run-time using a combination of forward and backward static analyses. The focus is on numeric properties of variables and numeric abstract domains, such as polyhedra. The obtained preconditions in the form of linear constraints are then analyzed to quantify how likely is an input to satisfy them. Model counting techniques are employed to count the number of solutions that satisfy given linear constraints. These counts are then used to assess the probability that the target assertion is satisfied/violated. We also present how to extend our approach to analyze non-deterministic programs by inferring sufficient preconditions. We built a prototype implementation and evaluate it on several interesting examples.

## 1 Introduction

Program verification is often concerned by only determining whether one assertion always holds at a given program point. However, there are many applications where we need to know a more fine-grained information about how likely a target assertion (event) is to be satisfied/violated. Examples of other target events include the invocation of a certain method, the access to confidential information, etc. In those cases, we want to distinguish between what is possible event (even with extremely low probability) and what is likely event (possible with higher probability). In this work, we show how to calculate the reliability of programs by using combination of static analysis by abstract interpretation and model counting. In particular, we are interested to learn how the presence of uncertainty in the inputs can affect the probability of assertions at the exit of the program.

This is an important problem to consider, since uncertainty is a common aspect of many real-world software systems today (e.g., medicine and aerospatial domains).

Abstract interpretation [6,7,8] is a general theory for approximating the semantics of programs. It provides safe (all answers are correct) and efficient (with a good trade-off between precision and cost) static analyses of run-time properties of real programs. It is based on the idea of *approximations* between concrete and abstract domains of program properties. Its practical success is mainly enabled by the design of numerical abstract domains, which reason on numerical properties of variables. For example, the interval domain [6], which is non-relational, infers the information about the possible values of individual variables; the octagon domain [25], which is weakly relational, infers unit binary linear constraints between program variables; and the polyhedra domain [10], which is fully relational, infers the linear constraints between all program variables. Abstract interpretation is a powerful technique for deriving approximate, albeit computable analyses, by using fully automatic algorithms. These abstract analyses pay the price for finite computability (always terminate) by an inevitable loss of precision. We use abstract analyses for automatic inference of (over-approximated) *invariants* by *forward analysis*, and (over-approximated) *necessary preconditions* by *backward analysis*. These two abstract analyses can be combined such that the results of the first analysis refine the results of the second one. In this work, we use a combination of forward and backward analyses to automatically generate the necessary preconditions on input variables that lead to the satisfaction/violation of a given assertion. If obtained preconditions are satisfied by some concrete values for input variables, then they represent input values that will allow the given assertion to be definitely satisfied/violated by all program executions branching from them. In fact, we run two backward analyses: the first one determines necessary preconditions for the given assertion to be satisfied, while the second one determines necessary preconditions for the given assertion to be violated.

*Model counting* is the problem of determining the number of solutions of a given constraint (formula). The LattE tool [1] implements state-of-the-art algorithms for computing volumes, both real and integral, of convex polytopes as well as integrating functions over those polytopes. More specifically, we use the LattE tool to estimate algorithmically the exact number of points of a bounded (possibly very large) discrete domain that satisfy given linear constraints.

In this paper, we describe a method which uses abstract interpretation-based static analysis and model counting to perform a specific type of quantitative analysis of deterministic programs, that is the calculation of *program reliability*. Calculating the program reliability involves counting the number of solutions to preconditions, which are given in the form of linear constraints between variables, i.e. elements from the polyhedra domain, that ensure satisfaction/violation of a given assertion by using model counting, and dividing it by the total space of values of the inputs. We assume that the input values are *uniformly distributed* within their finite discrete domain. Since the set of generated preconditions represents an over-approximation, we compute the reliability of programs as upper and lower bounds of exact probabilities that a given assertion is satisfied or violated. The reported uncertainty is due to the approximation inherent in abstract interpretation, which is introduced in order to obtain a scalable and fully automatic analysis.

The focus here is on programs whose input values range over finite discrete domains. Thus, we obtain a finite input domain and so we can use model counting algorithms to compute the required probabilities. We also restrict ourselves to the domain of linear integer arithmetic, since this is supported by LattE and the polyhedra numeric domain we use.

We also consider an extension of our approach to non-deterministic programs. For non-deterministic programs, sufficient and necessary preconditions no more coincide [26]. Sufficient preconditions ensure that the target invariant holds for all sequences of non-deterministic choices made at each execution step, whereas necessary preconditions ensure that the target invariant holds for at least one sequence of non-deterministic choices made at each execution step. In effect, increasing the non-determinism will reduce the set of sufficient preconditions and enlarge the set of necessary preconditions. Hence, for non-deterministic programs we construct backward analyses for inferring (under-approximated) sufficient preconditions that lead to the satisfaction/violation of a given assertion. The calculation of reliability is then similar to the one for deterministic programs.

We have developed a prototype probabilistic static analyzer which uses the APRON library [21] to implement numeric property domains and the LattE tool [1] to implement model counting algorithms. APRON provides a common high-level API to the most common numerical property domains, such as intervals, octagons, and polyhedra. We have implemented a combination of forward and backward analyses of deterministic (resp., non-deterministic) C programs for the automatic inference of *invariants* and *necessary* (resp. *sufficient*) *preconditions* in all program points. Our static analyzer has two components: (1) it computes the required preconditions in the input program point for a given assertion to be satisfied/violated, and (2) it then calls LattE to count the number of solutions of those preconditions and calculates the program reliability.

The main contributions of this work are:

- We demonstrate how to calculate the program reliability of deterministic and non-deterministic programs using static analysis by abstract interpretation and model counting.
- We develop a probabilistic static analyzer, which uses numerical property domains from the APRON library and the LattE model counting tool.
- Finally, we evaluate our method for probabilistic static analysis of C programs and show how to handle a set of small but compelling benchmarks.

## 2 Motivating Examples

Consider the program $P_1$ in Fig. 1. Suppose that the initial value of `i` ranges over the integer domain $[0, 19]$, and the initial value is independently and uniformly distributed across this range. When ($\mathtt{i} \geq 10$) the variable `k` is assigned to 12, otherwise `k` is assigned to 50. A forward invariant analysis will find the invariant

```
void   main() {
①:     int i:=[0,19]; l_input :
②:     int k:=0;
③:     if (i ≥ 10) k:=12; else k:=50;
l_final: assert (k ≤ 30);
       }
```

Fig. 1: The program $P_1$

```
void   main() {
①:     int j:=[0,9]; l_input :
②:     int i:=0;
③:     while (i < 100) {
④:         i:=i+1;
⑤:         j:=j+1; }
l_final: assert (j ≤ 105);
       }
```

Fig. 2: The program $P_2$

$\mathtt{k} = 12 \vee \mathtt{k} = 50$ at point $l_{\mathtt{final}}$. Therefore, the assertion ($\mathtt{k} \leq 30$) can be satisfied (when $\mathtt{k} = 12$) and can be violated (when $\mathtt{k} = 50$). We are interested in inferring necessary preconditions on the input state at control point $l_{\mathtt{input}}$, when the assertion is satisfied and when the assertion is violated. We back-propagate necessary conditions of satisfaction and violation of the assertion from point $l_{\mathtt{final}}$ to $l_{\mathtt{input}}$. A backward necessary condition analysis will infer the precondition $\mathtt{i} \geq 10$ at point $l_{\mathtt{input}}$ assuming that the assertion is satisfied, and the precondition $\mathtt{i} < 10$ at point $l_{\mathtt{input}}$ assuming that the assertion is violated. The size of the input domain is 20, since $\mathtt{i} \in [0, 19]$. By calling LattE to count the number of solutions to the above preconditions, we can calculate that the probability for the assertion to be satisfied (*success probability*) is: $\frac{10}{20} = 50\%$, and the probability for the assertion to be violated (*failure probability*) is: $\frac{10}{20} = 50\%$.

Consider the program $P_2$ in Fig. 2. A forward invariant analysis will find the invariant $100 \leq \mathtt{j} \leq 109$ at point $l_{\mathtt{final}}$, so the corresponding assertion can be satisfied (when $100 \leq \mathtt{j} \leq 105$) and can be violated (when $105 < \mathtt{j} \leq 109$). A backward necessary condition analysis will infer the precondition $0 \leq \mathtt{j} \leq 5$ at point $l_{\mathtt{input}}$ for the assertion to be satisfied, and the precondition $5 < \mathtt{j} \leq 9$ at point $l_{\mathtt{input}}$ for the assertion to be violated. Therefore, we can calculate that the *success probability* is: $\frac{6}{10} = 60\%$, and the *failure probability* is: $\frac{4}{10} = 40\%$.

## 3 Forward-Backward Precondition Analyses

We describe the combination of forward and backward analyses in the framework of abstract interpretation for inferring necessary preconditions that a given assertion is satisfied/violated. The principle of the combination is to use the result of the forward invariant analysis in the subsequent backward necessary condition analysis in order to get more precise results which are still sound.

*Syntax.* We consider a simple deterministic programming language that is a subset of C, which will be used to exemplify our work. The control point (location) before each statement and at the end of each block is associated to a unique label $l \in \mathbb{L}$. The syntax of the language is given by:

$$s ::= \mathtt{skip} \mid \mathtt{x:=}e \mid \mathtt{x:=}[n, n'] \mid s\,;s \mid \mathsf{if}\ (e)\ \mathsf{then}\ s\ \mathsf{else}\ s \mid \mathtt{while}\ (e)\ \mathtt{do}\ s \mid \mathtt{assert}(e)$$

$$e ::= n \mid \mathtt{x} \mid e \oplus e$$

where $n$ ranges over integers, $[n, n']$ ranges over integer intervals, $\mathtt{x}$ ranges over variable names *Var*, and $\oplus$ over arithmetic-logic operators. Non-deterministic interval assignment $\mathtt{x}\mathtt{:=}[n, n']$ represents an input statement which assigns to the input variable $\mathtt{x}$ a uniformly distributed random value from the interval $[n, n']$. This interval assignment can occur only in the *input section* of the program, and is used to model input uncertainties. The set of all generated statements $s$ is denoted by *Stm*, whereas the set of all expressions $e$ is denoted by *Exp*. We assume $l_{\mathtt{input}}$ is the location after the input statements (i.e. it denotes the end of the input section) and $l_{\mathtt{final}}$ is the location at the end of the program, where an assertion $\mathtt{assert}(e_{\mathtt{f}})$ is posed. Without loss of generality, a program is a sequence of statements followed by a single assertion.

*Concrete semantics.* A *program state* is given by a control location in $\mathbb{L}$ and an environment in $\mathcal{E} : Var \to \mathbb{Z}$ mapping each variable to its value (integer number). We write $\Sigma = \mathbb{L} \times \mathcal{E}$ to denote the set of all possible program states. Programs are modelled as transition systems $(\Sigma, \longrightarrow)$, where $\Sigma$ is a set of states and $\longrightarrow \subseteq \Sigma \times \Sigma$ is a transition relation modelling atomic execution steps. The relation $\longrightarrow$ is defined by local rules, such as the following:

**assignment** $l_0 : \mathtt{x}\mathtt{:=}e; l_1$ **::** $(l_0, \rho) \longrightarrow (l_1, \rho[\mathtt{x} \mapsto [\![e]\!](\rho)])$, where $[\![e]\!](\rho) \in \mathbb{Z}$ is the result of the evaluation of $e$ in the environment $\rho$, and $\rho[\mathtt{x} \mapsto n]$ denotes the environment that updates $\rho$ at variable $\mathtt{x}$ with the value $n$.

**input** $l_0 : \mathtt{x}\mathtt{:=}[n, n']; l_1$ **::** $(l_0, \rho) \longrightarrow (l_1, \rho[\mathtt{x} \mapsto n''])$, where $n'' \in [n, n']$.

**conditional** $l_0$ : $\mathsf{if}$ $(e)$ $\mathsf{then}$ $\{l_0^t : s;\ l_1^t\}$ $\mathsf{else}$ $\{l_0^f : s';\ l_1^f\}; l_1$ **::** $(l_0, \rho) \longrightarrow (l_0^t, \rho)$ if $[\![e]\!](\rho) \neq 0$ [3], $(l_0, \rho) \longrightarrow (l_0^f, \rho)$ if $[\![e]\!](\rho) = 0$, $(l_1^t, \rho) \longrightarrow (l_1, \rho)$, and $(l_1^f, \rho) \longrightarrow (l_1, \rho)$.

**loop** $l_0 : \mathtt{while}$ $(e)$ $\mathtt{do}$ $\{l_0^t : s;\ l_1^t\}; l_1$ **::** $(l_0, \rho) \longrightarrow (l_0^t, \rho)$ if $[\![e]\!](\rho) \neq 0$, $(l_0, \rho) \longrightarrow (l_1, \rho)$ if $[\![e]\!](\rho) = 0$, and $(l_1^t, \rho) \longrightarrow (l_0, \rho)$. [4]

Let $\mathbb{E} \subseteq \mathcal{E}$ be the set of input environments obtained after executing the input statements. The set of input states is $\mathcal{I} = \{(l_{\mathtt{input}}, \rho) \mid \rho \in \mathbb{E}\}$. The invariant inference (reachability) problem consists of finding out the possible environments (values of all variables) that may arise at each control location. The concrete semantic domain is the complete lattice of the powerset of states $(\mathcal{P}(\Sigma), \subseteq, \cup, \cap, \emptyset, \Sigma)$, and the concrete semantics in the form of invariant states encountered branching from $\mathcal{I}$, denoted $\mathtt{inv}(\mathcal{I})$, is:

$$\mathtt{inv}(\mathcal{I}) = \mathtt{lfp}_{\mathcal{I}} \lambda X. X \cup \mathtt{post}(X)$$

where $\mathtt{post}(X) = \{\sigma \in \Sigma \mid \exists \sigma' \in X. \sigma' \longrightarrow \sigma\}$ and $\mathtt{lfp}_{\mathcal{I}} f$ is the least fixed point of the function $f$ greater than $\mathcal{I}$.

In this work, we consider the problem of inferring necessary preconditions. Assume that a program exits with $l_{\mathtt{final}} : \mathtt{assert}(e_{\mathtt{f}})$. We want to distinguish

[3] Following the convention popularized by C, we model Boolean values as integers, with zero interpreted as false and everything else as true.

[4] Note that control moves from the final label $l_1^t$ of $s$ to the initial label $l_0$ of $\mathtt{while}$.

between program termination that leads to the satisfaction of the final assertion at $l_{\mathtt{final}}$ from the one that leads to the violation of the final assertion at $l_{\mathtt{final}}$. Let $\mathcal{F}_{sat} = \{(l,\rho) \in \mathtt{inv}(\mathcal{I}) \mid l = l_{\mathtt{final}} \implies [\![e_{\mathtt{f}}]\!](\rho) \neq 0\}$ and $\mathcal{F}_{viol} = \{(l,\rho) \in \mathtt{inv}(\mathcal{I}) \mid l = l_{\mathtt{final}} \implies [\![e_{\mathtt{f}}]\!](\rho) = 0\}$ be the invariant sets which enforce the assertion at the point $l_{\mathtt{final}}$ to be satisfied and violated, respectively, and coincide with $\mathtt{inv}(\mathcal{I})$ everywhere else. In the following, $\mathcal{F}$ may represent either $\mathcal{F}_{sat}$ or $\mathcal{F}_{viol}$. Given an invariant set $\mathcal{F}$ to obey, we want to infer the set of input states $\mathtt{cond}(\mathcal{F})$ that guarantee that all program executions stay in $\mathcal{F}$:

$$\mathtt{cond}(\mathcal{F}) = \mathtt{gfp}_{\mathcal{F}} \lambda X. X \cap \mathtt{pre}(X)$$

where $\mathtt{pre}(X) = \{\sigma \in \Sigma \mid \exists \sigma' \in X. \sigma \longrightarrow \sigma'\}$ is the set of predecessors of $X$, and $\mathtt{gfp}_{\mathcal{F}} f$ is the greatest fixed point of the function $f$ smaller than $\mathcal{F}$. The above two fixed points ($\mathtt{lfp}$ and $\mathtt{gfp}$) exist according to Tarski, as the corresponding functions are monotone and continuous in the complete lattice of state sets.

Given a set of input environments $\mathbb{E} \subseteq \mathcal{E}$, we can compute the subsets $\mathbb{E}_{sat}$ and $\mathbb{E}_{viol}$ of input environments that lead to satisfaction and violation of the final assertion as:

$$\mathbb{E}_{sat} = \mathbb{E} \cap \{\rho \mid (l_{\mathtt{input}}, \rho) \in \mathtt{cond}(\mathcal{F}_{sat})\}, \mathbb{E}_{viol} = \mathbb{E} \cap \{\rho \mid (l_{\mathtt{input}}, \rho) \in \mathtt{cond}(\mathcal{F}_{viol})\}$$

*Abstract semantics.* Transition systems can become large or infinite for real programs, so that neither $\mathtt{inv}(\mathcal{I})$ nor $\mathtt{cond}(\mathcal{F})$ can be computed at all. Therefore, we seek for sound approximations. The actual computable abstract analyses can be defined as over-approximations of the concrete semantics. A static analyzer will infer over-approximated necessary preconditions so that all program executions that lead to satisfaction (resp., violation) of the final assertion are taken into account, thus computing an over-approximation of $\mathbb{E}_{sat}$ (resp., $\mathbb{E}_{viol}$).

We consider an abstract domain $(\mathbb{D}, \sqsubseteq_{\mathbb{D}})$, such that there exist a Galois connection [5] $\langle \mathcal{P}(\mathcal{E}), \subseteq \rangle \underset{\alpha_{\mathbb{D}}}{\overset{\gamma_{\mathbb{D}}}{\leftrightarrows}} \langle \mathbb{D}, \sqsubseteq_{\mathbb{D}} \rangle$. We assume that the abstract domain $\mathbb{D}$ is equipped with sound operators for ordering $\sqsubseteq_{\mathbb{D}}$, least upper bound (join) $\sqcup_{\mathbb{D}}$, greatest lower bound (meet) $\sqcap_{\mathbb{D}}$, bottom $\bot_{\mathbb{D}}$, top $\top_{\mathbb{D}}$, widening $\nabla_{\mathbb{D}}$, and narrowing $\triangle_{\mathbb{D}}$, as well as sound transfer functions for assignments $\overrightarrow{\mathtt{assign}}_{\mathbb{D}} : \mathit{Var} \times \mathit{Exp} \times \mathbb{D} \to \mathbb{D}$, tests $\overrightarrow{\mathtt{filter}}_{\mathbb{D}} : \mathit{Exp} \times \mathbb{D} \to \mathbb{D}$, and backward assignments $\overleftarrow{\mathtt{b\text{-}assign}}_{\mathbb{D}} : \mathit{Var} \times \mathit{Exp} \times \mathbb{D} \times \mathbb{D} \to \mathbb{D}$. We let $\mathtt{lfp}^{\#}$ (resp., $\mathtt{gfp}^{\#}$) denote an abstract post-fixpoint (resp., pre-fixpoint) operator, derived using widening $\nabla_{\mathbb{D}}$ and narrowing $\triangle_{\mathbb{D}}$, that over-approximates the concrete $\mathtt{lfp}$ (resp., $\mathtt{gfp}$) [8]. Finally, the concrete domain on which concrete semantics is defined $(\mathcal{P}(\Sigma), \subseteq)$ is abstracted using a Galois connection $\langle \mathcal{P}(\Sigma), \subseteq \rangle \underset{\alpha}{\overset{\gamma}{\leftrightarrows}} \langle \mathbb{L} \to \mathbb{D}, \dot{\sqsubseteq} \rangle$ where $\alpha(R) = \lambda l \in \mathbb{L}. \sqcup_{\mathbb{D}} \{d \in \mathbb{D} \mid (l,\rho) \in R, \alpha_{\mathbb{D}}(\rho) = d\}$. Hence, each control point $l \in \mathbb{L}$ is associated with an element $d \in \mathbb{D}$ in the abstract semantics.

[5] $\langle L, \leq_L \rangle \underset{\alpha}{\overset{\gamma}{\leftrightarrows}} \langle M, \leq_M \rangle$ is a *Galois connection* between complete lattices $L$ and $M$ iff $\alpha$ and $\gamma$ are total functions that satisfy: $\alpha(l) \leq_M m \iff l \leq_L \gamma(m)$ for all $l \in L, m \in M$. Here $\leqslant_L$ and $\leqslant_M$ are the pre-order relations for $L$ and $M$, respectively.

We define a family of forward transfer functions $\overrightarrow{\delta}_{l,l'} : \mathbb{D} \to \mathbb{D}$ that compute the effect of any concrete transition at the abstract level. The definition of $\overrightarrow{\delta}_{l,l'}$ for some statements is:

**assignment** $l_0 : \texttt{x:=}e; l_1$ **::** $\overrightarrow{\delta}_{l_0,l_1}(d) = \overrightarrow{\texttt{assign}_{\mathbb{D}}}(\texttt{x}, e, d))$.
**conditional** $l_0$ : if $(e)$ then $\{l_0^t : s;\ l_1^t\}$ else $\{l_0^f : s';\ l_1^f\}; l_1$ **::** $\overrightarrow{\delta}_{l_0,l_0^t}(d) = \overrightarrow{\texttt{filter}_{\mathbb{D}}}(e, d)$, $\overrightarrow{\delta}_{l_0,l_0^f}(d) = \overrightarrow{\text{filter}_{\mathbb{D}}}(\neg e, d)$, $\overrightarrow{\delta}_{l_1^t,l_1}(d) = d$, $\overrightarrow{\delta}_{l_1^f,l_1}(d) = d$.
**loop** $l_0 : \texttt{while}\ (e)\ \texttt{do}\ \{l_0^t : s;\ l_1^t\}; l_1$ **::** $\overrightarrow{\delta}_{l_0,l_0^t}(d) = \overrightarrow{\texttt{filter}_{\mathbb{D}}}(e, d)$, $\overrightarrow{\delta}_{l_0,l_1}(d) = \overrightarrow{\text{filter}_{\mathbb{D}}}(\neg e, d)$, and $\overrightarrow{\delta}_{l_1^t,l_0}(d) = d$.

The soundness of $\{\overrightarrow{\delta}_{l,l'} \mid l, l' \in \mathbb{L}\}$ is written as: $\forall d \in \mathbb{D}, \forall \rho \in \gamma_{\mathbb{D}}(d), (l, \rho) \longrightarrow (l', \rho') \implies \rho' \in \gamma_{\mathbb{D}}(\overrightarrow{\delta}_{l,l'}(d))$.

Suppose that the abstract element $\alpha_{\mathbb{D}}(\mathbb{E}) = d_{\texttt{input}} \in \mathbb{D}$ is at the input control point $l_{\texttt{input}}$. We can collect the abstractions of possible environments at each program control point using the following forward interpreter:

$$\overrightarrow{F}^{\#} = \lambda I.\lambda(l \in \mathbb{L}). \sqcup_{\mathbb{D}} \{\overrightarrow{\delta}_{l',l}(I(l')) \mid l' \in \mathbb{L}\}$$

such that the result of the forward analyzer is $\overrightarrow{\mathcal{I}}^{\#} = \texttt{lfp}^{\#}_{I_0} \overrightarrow{F}^{\#}$, where $I_0(l_{\texttt{input}}) = d_{\texttt{input}}$. Assume that $\texttt{lfp}^{\#}_{I_0} \overrightarrow{F}^{\#}(l_{\texttt{final}}) = d_{\texttt{final}}$. Let $d^{sat}_{\texttt{final}} = \overrightarrow{\text{filter}_{\mathbb{D}}}(e, d_{\texttt{final}})$ and $d^{viol}_{\texttt{final}} = \overrightarrow{\text{filter}_{\mathbb{D}}}(\neg e, d_{\texttt{final}})$. We want to design two backward abstract interpreters that propagate backwards the invariants ensuring that the final assertion is satisfied $d^{sat}_{\texttt{final}}$ and violated $d^{viol}_{\texttt{final}}$, respectively. The backward interpreters refine the invariants found by $\overrightarrow{F}^{\#}$. Thus, they take two elements of $\mathbb{D}$ as inputs: an invariant to refine and an invariant to propagate backwards. They are based on a family of backward transfer functions $\overleftarrow{\delta}_{l,l'} : \mathbb{D} \times \mathbb{D} \to \mathbb{D}$, which map a precondition to refine and a postcondition into a refined precondition. The definition of $\overleftarrow{\delta}_{l,l'}$ for some statements is:

**assignment** $l_0 : \texttt{x:=}e; l_1$ **::** $\overleftarrow{\delta}_{l_0,l_1}(d, d') = \overleftarrow{\texttt{b-assign}_{\mathbb{D}}}(\texttt{x}, e, d, d')$.
**conditional** $l_0$ : if $(e)$ then $\{l_0^t : s;\ l_1^t\}$ else $\{l_0^f : s';\ l_1^f\}; l_1$ **::** $\overleftarrow{\delta}_{l_0,l_0^t}(d, d') = d \sqcap d'$, $\overleftarrow{\delta}_{l_0,l_0^f}(d, d') = d \sqcap d'$, $\overleftarrow{\delta}_{l_1^t,l_1}(d, d') = d \sqcap d'$, $\overleftarrow{\delta}_{l_1^f,l_1}(d, d') = d \sqcap d'$.
**loop** $l_0 : \texttt{while}\ (e)\ \texttt{do}\ \{l_0^t : s;\ l_1^t\}; l_1$ **::** $\overleftarrow{\delta}_{l_0,l_0^t}(d, d') = d \sqcap d'$, $\overleftarrow{\delta}_{l_0,l_1}(d, d') = d \sqcap d'$, and $\overleftarrow{\delta}_{l_1^t,l_0}(d, d') = d \sqcap d'$.

The soundness of $\{\overleftarrow{\delta}_{l,l'} \mid l, l' \in \mathbb{L}\}$ is written as: $\forall d, d' \in \mathbb{D}, \forall \rho \in \gamma_{\mathbb{D}}(d), \rho' \in \gamma_{\mathbb{D}}(d'), (l, \rho) \longrightarrow (l', \rho') \implies \rho \in \gamma_{\mathbb{D}}(\overleftarrow{\delta}_{l,l'}(d, d'))$. That is, $d$ is refined into a stronger precondition by taking into account the postcondition $d'$.

Suppose that $F^{sat}_{\mathbb{D}}(l_{\texttt{final}}) = d^{sat}_{\texttt{final}}$, $F^{viol}_{\mathbb{D}}(l_{\texttt{final}}) = d^{viol}_{\texttt{final}}$, and $F^{sat}_{\mathbb{D}}(l) = F^{viol}_{\mathbb{D}}(l) = \overrightarrow{\mathcal{I}}^{\#}(l)$ for $l \neq l_{\texttt{final}}$. The backward interpreters are defined as:

$$\overleftarrow{F}^{\#} = \lambda(I, F).\lambda(l \in \mathbb{L}). \sqcap_{\mathbb{D}} \{\overleftarrow{\delta}_{l,l'}(I(l), F(l')) \mid l' \in \mathbb{L}\}$$

such that the results of the two backward analyzers are: $\overleftarrow{C}^{\#}_{sat} = \texttt{gfp}^{\#}_{(\overrightarrow{\mathcal{I}}^{\#}, F^{sat}_{\mathbb{D}})} \overleftarrow{F}^{\#}$ and $\overleftarrow{C}^{\#}_{viol} = \texttt{gfp}^{\#}_{(\overrightarrow{\mathcal{I}}^{\#}, F^{viol}_{\mathbb{D}})} \overleftarrow{F}^{\#}$. The necessary preconditions that the final assertion is satisfied and violated are $d^{sat}_{\texttt{input}} = \overleftarrow{C}^{\#}_{sat}(l_{\texttt{input}})$ and $d^{viol}_{\texttt{input}} = \overleftarrow{C}^{\#}_{viol}(l_{\texttt{input}})$, respectively. We can now compute the over-approximated sets $\mathbb{E}^{\#}_{sat}$ and $\mathbb{E}^{\#}_{viol}$ of input environments $\mathbb{E}_{sat}$ and $\mathbb{E}_{viol}$ that lead to satisfaction and violation of the final assertion as:

$$\mathbb{E}^{\#}_{sat} = \mathbb{E} \cap \gamma_{\mathbb{D}}(d^{sat}_{\texttt{input}}), \quad \mathbb{E}^{\#}_{viol} = \mathbb{E} \cap \gamma_{\mathbb{D}}(d^{viol}_{\texttt{input}})$$

such that $\mathbb{E}^{\#}_{sat} \supseteq \mathbb{E}_{sat}$ and $\mathbb{E}^{\#}_{viol} \supseteq \mathbb{E}_{viol}$.

*Polyhedra numeric abstract domain.* Although, the abstract domain $\mathbb{D}$ can be instantiated with different property domains, in the following, we will use the polyhedra numerical abstract domain for $\mathbb{D}$. This is due to the fact that only for the polyhedra domain all necessary abstract operations and transfer functions, such as $\overrightarrow{\texttt{assign}}_{\mathbb{D}}$, $\overleftarrow{\texttt{b-assign}}_{\mathbb{D}}$, $\overleftarrow{\texttt{b-assign-under}}_{\mathbb{D}}$ (see Section 5), are implemented in the APRON library. The *Polyhedra domain* [10], denoted as $\langle P, \sqsubseteq_P \rangle$, is a fully relational numerical property domain, which allows manipulating conjunctions of linear inequalities of the form $\alpha_1 \texttt{x}_1 + \ldots + \alpha_n \texttt{x}_n \geq \beta$, where $\texttt{x}_1, \ldots, \texttt{x}_n$ are program variables and $\alpha_i, \beta \in \mathbb{R}$ (reals). The abstract operations of the *Polyhedra domain* are defined in [10]. Polyhedra analysis is expensive but also very precise.

A property element is represented as a conjunction of linear constraints given in the matrix form $\langle \mathbf{A}, \mathbf{b} \rangle$ that consists of a matrix $\mathbf{A} \in \mathbb{R}^{m \times n}$ and a vector $\mathbf{b} \in \mathbb{R}^m$, where $n$ is the number of variables and $m$ is the number of constraints. This is called the constraint representation of polyhedra elements, and there is another so-called generator representation. One representation can be converted to the other one using the Chernikova's algorithm [5]. Some domain operations can be performed more efficiently using the generator representation only, others based on the constraint representation, and some making use of both. We now present some operations that can be defined using the constraint representation.

The concretization function is: $\gamma_P(\langle \mathbf{A}, \mathbf{b} \rangle) = \{\mathbf{v} \in \mathbb{R}^n \mid \mathbf{A} \cdot \mathbf{v} \geq \mathbf{b}\}$. The meet $\sqcap_P$ is defined as: $\langle \mathbf{A}_1, \mathbf{b_1} \rangle \sqcap_P \langle \mathbf{A}_2, \mathbf{b_2} \rangle = \langle \binom{\mathbf{A}_1}{\mathbf{A}_2}, \binom{\mathbf{b_1}}{\mathbf{b_2}} \rangle$. We also need widening since the polyhedra domain has infinite strictly increasing chains.

$$\langle \mathbf{A}_1, \mathbf{b_1} \rangle \nabla_P \langle \mathbf{A}_2, \mathbf{b_2} \rangle = \{c \in \langle \mathbf{A}_1, \mathbf{b_1} \rangle \mid \langle \mathbf{A}_2, \mathbf{b_2} \rangle \sqsubseteq_P \{c\}\}$$

where $c$ represents one constraint from $\langle \mathbf{A}_1, \mathbf{b_1} \rangle$. The transfer function $\overrightarrow{\texttt{filter}}_P$ abstracts affine inequality expressions by adding them to the input polyhedra.

$$\overrightarrow{\texttt{filter}}_P(\sum_i \alpha_i \texttt{x}_i \geq \beta, \langle \mathbf{A}, \mathbf{b} \rangle) = \langle \begin{pmatrix} \mathbf{A} \\ \alpha_1 \ldots \alpha_n \end{pmatrix}, \begin{pmatrix} \mathbf{b} \\ \beta \end{pmatrix} \rangle$$

*Example 1.* Consider the program $P_1$ from Fig. 1. Assume that $\mathbb{D}$ is the polyhedra domain. The input abstract element is $d_{\texttt{input}} = (0 \leq \texttt{i} \leq 19)$. Using the forward analyzer $\overrightarrow{F}^{\#}$, we obtain $d_{\texttt{final}} = (\texttt{k} = 12 \vee \texttt{k} = 50)$, and so $d^{sat}_{\texttt{final}} = (\texttt{k} = 12)$ and $d^{viol}_{\texttt{final}} = (\texttt{k} = 50)$. Using backward analyzers $\overleftarrow{F}^{\#}$, we obtain $d^{sat}_{\texttt{input}} = (\texttt{i} \geq 10)$ and $d^{viol}_{\texttt{input}} = (\texttt{i} < 10)$. □

## 4 Computing Success and Failure Probabilities

The overall goal of our approach is to answer questions about the probability of assertions at the exit of a deterministic program $P$. We define the *success probability* as the probability that a program terminates successfully with the target assertion being satisfied. The *failure probability* is the probability that a program hits a failure caused by the target assertion being violated.

The combination of forward and two backward analyses infers the necessary preconditions, denoted $d^{sat}_{\texttt{input}} = \overleftarrow{C}^{\#}_{sat}(l_{\texttt{input}})$ and $d^{viol}_{\texttt{input}} = \overleftarrow{C}^{\#}_{viol}(l_{\texttt{input}})$, that the target assertion is satisfied and violated, respectively. Calculating the likelihood of satisfying/violating the given assertion involves counting the number of solutions to $d^{sat}_{\texttt{input}}/d^{viol}_{\texttt{input}}$ and dividing it by the total space of possible values in its input domain $\mathbb{E}$. In particular, we use model counting techniques and LattE tool [1] to estimate algorithmically the exact number of points of a bounded (possibly very large) discrete domain $\mathbb{E}$ that satisfy the (linear) constraints $d^{sat}_{\texttt{input}}$ and $d^{viol}_{\texttt{input}}$. We restrict our attention on programs that have *finite input domains* $\mathbb{E}$ and on numeric abstract elements from the polyhedra domain expressed as *linear integer arithmetic* (LIA) constraints over program variables whose values are *uniformly distributed* over their input domain.

We use the LattE tool to compute the number of elements of $\mathbb{E}$ that satisfy $d^{sat}_{\texttt{input}}$ and $d^{viol}_{\texttt{input}}$, denoted $\#(d^{sat}_{\texttt{input}})$ and $\#(d^{viol}_{\texttt{input}})$. The size of $\mathbb{E}$, denoted $\#(\mathbb{E})$, is the product of domain's sizes of all input variables in program $P$. Thus, we have: $\#(\mathbb{E}) = \prod_{\texttt{x:=}[n,n']\in P} |n'-n+1|$. Note that the exact sets of input states that lead to satisfaction and violation of the given assertion are $\mathbb{E}_{sat}$ and $\mathbb{E}_{viol}$, and their sizes are denoted $\#(\mathbb{E}_{sat})$ and $\#(\mathbb{E}_{viol})$. Since the found necessary preconditions $d^{sat}_{\texttt{input}}$ and $d^{viol}_{\texttt{input}}$ are over-approximations of $\mathbb{E}_{sat}$ and $\mathbb{E}_{viol}$ respectively, we have $\#(\mathbb{E}_{sat}) \leq \#(d^{sat}_{\texttt{input}})$ and $\#(\mathbb{E}_{viol}) \leq \#(d^{viol}_{\texttt{input}})$. Moreover, the input environments which are not in $\gamma_{\mathbb{D}}(d^{sat}_{\texttt{input}})$, that is they are in $\mathbb{E}\backslash\gamma_{\mathbb{D}}(d^{sat}_{\texttt{input}})$, definitely lead to the violation of the assertion. Therefore, we have $\#(\mathbb{E}) - \#(d^{sat}_{\texttt{input}}) \leq \#(\mathbb{E}_{viol}) \leq \#(d^{viol}_{\texttt{input}})$. By similar reasoning as above, we can also establish that: $\#(\mathbb{E}) - \#(d^{viol}_{\texttt{input}}) \leq \#(\mathbb{E}_{sat}) \leq \#(d^{sat}_{\texttt{input}})$. Finally, we calculate the *success* and *failure probability* of a program $P$ as follows:

$$\begin{array}{c} \frac{\#(\mathbb{E})-\#(d^{viol}_{\texttt{input}})}{\#(\mathbb{E})} \leq \mathbf{Pr^s(P)} = \frac{\#(\mathbb{E}_{sat})}{\#(\mathbb{E})} \leq \frac{\#(d^{sat}_{\texttt{input}})}{\#(\mathbb{E})} \\ \frac{\#(\mathbb{E})-\#(d^{sat}_{\texttt{input}})}{\#(\mathbb{E})} \leq \mathbf{Pr^f(P)} = \frac{\#(\mathbb{E}_{viol})}{\#(\mathbb{E})} \leq \frac{\#(d^{viol}_{\texttt{input}})}{\#(\mathbb{E})} \end{array} \quad (1)$$

Note that $Pr^s(P) + Pr^f(P) = 1$.

*Example 2.* Consider the program $P_1$ from Fig. 1. We have $\mathbb{E} = \{[\texttt{i} \mapsto n] \mid n \in [0, 19]\}$, and so $\#(\mathbb{E}) = 20$. Using forward and two backward analyses, we obtain $d^{sat}_{\texttt{input}} = (\texttt{i} \geq 10)$ and $d^{viol}_{\texttt{input}} = (\texttt{i} < 10)$, and so $\#(d^{sat}_{\texttt{input}}) = 10$ and $\#(d^{viol}_{\texttt{input}}) = 10$. Thus, the success and failure probabilities are:

$$Pr^s(P_1) = \frac{10}{20}\,(50\%), \quad \text{and} \quad Pr^f(P_1) = \frac{10}{20}\,(50\%) \qquad \square$$

We use model counting and the LattE tool [1] to determine the number of solutions of a given constraint. LattE accepts LIA constraints expressed as a system of linear inequalities each of which defines a hyperplane encoded as the matrix inequality: $Ax \leq B$, where $A$ is an $m \times n$ matrix of coefficients and $B$ is an $m \times 1$ column vector of constants. Most LIA constraints can easily be converted into the form: $a_1x_1 + \ldots + a_nx_n \leq b$. For example, $\geq$ and $>$ can be flipped by multiplying both sides by $-1$, and strict inequalities $<$ can be converted by decrementing the constant $b$. In LattE, equalities $=$ can be expressed directly. If we have disequalities $\neq$, they can be handled by counting a set of constraints that encode all possible solutions. For example, the constraint $\alpha \wedge (x_1 \neq x_2)$ is handled by finding the sum of solutions for $\alpha \wedge (x_1 \leq x_2 - 1)$ and $\alpha \wedge (x_1 \geq x_2 + 1)$. For a system $Ax \leq B$, where $A$ is an $m \times n$ matrix and $B$ is an $m \times 1$ column vector, the input LattE file is:

$$\begin{array}{cc} m & n{+}1 \\ B & -A \end{array}$$

where the first line indicates the matrix size: the number of inequalities $m$ by the number of variables $n$ plus one. The following lines encode all inequalities.

## 5 Extension to non-deterministic programs

Let us reconsider the program $P_2$ from Fig. 2, where the assignment in point ⑤ is now replaced with: `j:=j+[0,1]`. That is, the variable `j` is incremented by a uniformly distributed random integer between 0 and 1 at each iteration. We denote this non-deterministic program as $P_3$ (taken from [26]), given below:

```
void  main() {
①:    int j:=[0,9]; l_input :
②:    int i:=0;
③:    while (i < 100) {
④:        i:=i+1;
⑤:        j:=j+[0,1]; }
l_final : assert (j ≤ 105); }
```

A forward invariant analysis will find that at $l_{\texttt{final}}$ holds: $0 \leq \texttt{j} \leq 109$, and so the assertion ($\texttt{j} \leq 105$) can be both satisfied and violated. A backward necessary condition analysis for assertion satisfaction will infer the precondition $0 \leq \texttt{j} \leq 9$ at $l_{\texttt{input}}$, since for any value $\texttt{j} \in [0, 9]$ there exists an program execution satisfying the assertion (e.g., consider the executions where the random integer from $[0, 1]$ always evaluates to 0 in the body of `while`). However, a *backward sufficient condition analysis* for assertion satisfaction computes the set of input states such that all program executions branching from them satisfy the assertion. In this case, the sufficient condition analysis will infer the precondition $0 \leq \texttt{j} \leq 5$ at $l_{\texttt{input}}$, since even if the random integer from $[0, 1]$ always evaluates to 1 in the body of `while`, the assertion will always hold. As a result of this, we can conclude that the success probability is greater or equal to: $\frac{6}{10} = 60\%$.

We can see that necessary and sufficient preconditions are different in the presence of non-determinism [26]. Note that, if the non-determinism is increased in a program, then the set of sufficient preconditions will be reduced, while the set of necessary preconditions will be enlarged. For non-deterministic programs, $\mathbb{E}_{sat}$ and $\mathbb{E}_{viol}$ are subsets of input environments $\mathbb{E}$ that definitely lead to satisfaction and violation of the final assertion for all possible non-deterministic choices, respectively. We define the *success probability* $Pr^s(P)$ as the probability that a program terminates successfully with the target assertion being satisfied for all possible non-deterministic choices taken at each step. The *failure probability* $Pr^f(P)$ is the probability that a program hits a failure caused by the target assertion being violated for all possible non-deterministic choices taken at each step. We now show how to compute the success and failure probabilities for non-deterministic programs using sufficient conditions.

*Remark.* Note that in case of deterministic programs, $\mathbb{E}_{sat}$ and $\mathbb{E}_{viol}$ form a partition of the set of input environments $\mathbb{E}$ ($\mathbb{E}_{sat} \cup \mathbb{E}_{viol} = \mathbb{E}$), thus we have $Pr^s(P) + Pr^f(P) = 1$ for any deterministic program $P$. However, for non-deterministic programs this is not true anymore. That is, $\mathbb{E}_{sat} \cup \mathbb{E}_{viol} \subseteq \mathbb{E}$ and $Pr^s(P) + Pr^f(P) \leq 1$ for any non-deterministic program $P$. This means that there exist input environments for which it is possible the target assertion to be both satisfied and violated depending on non-deterministic choices made at each step of the given execution. For example, in the above program $P_3$, for input environments that satisfy $6 \leq \mathtt{j} \leq 9$, the target assertion is satisfied (when $[0, 1]$ in the body of `while` always evaluates to 0) and violated (when $[0, 1]$ in the body of `while` always evaluates to 1), so those input environments are neither in $\mathbb{E}_{sat}$ nor in $\mathbb{E}_{viol}$. We have, $\mathbb{E}_{sat} = \{\rho \mid 0 \leq [\![\mathtt{j}]\!]\rho \leq 5\}$ and $\mathbb{E}_{viol} = \emptyset$, thus $Pr^s(P_3) = 60\%$ and $Pr^f(P_3) = 0\%$.

*Syntax.* The extended non-deterministic programming language includes the same expression and statement productions as previously (see Section 3), but we add a support for non-deterministic expressions by using integer intervals $[n, n']$:

$$e ::= \ \dots \mid [n, n']$$

The integer interval $[n, n']$ denotes a uniformly distributed random integer from the interval $[n, n']$ (non-deterministic choice of an integer). Note that the interval assignment $\mathtt{x}\!:=\![n, n']$ can now be freely used everywhere in programs, not only in the input section as in deterministic programs.

*Concrete semantics.* We now consider the problem of backward sufficient condition inference. Given an invariant set $\mathcal{F}$ to obey, we want to infer the set of input states $\mathtt{cond}(\mathcal{F})$ that guarantee that all program executions branching from them for all possible non-deterministic choices taken at each step stay in $\mathcal{F}$:

$$\mathtt{cond}(\mathcal{F}) = \mathtt{gfp}_{\mathcal{F}} \lambda X. X \cap \widetilde{\mathtt{pre}}(X)$$

where $\widetilde{\mathtt{pre}}(X) = \{\sigma \in \Sigma \mid \forall \sigma' \in \Sigma. \sigma \longrightarrow \sigma' \implies \sigma' \in X\}$ is the set of states which represent predecessors only of states in $X$. Note that the function $\widetilde{\mathtt{pre}}(X)$

differs from the function $\texttt{pre}(X)$ used in Section 3, that is $\widetilde{\texttt{pre}}(X) \neq \texttt{pre}(X)$, if the transition system is non-deterministic (i.e. some states have several successors or none). Using $\widetilde{\texttt{pre}}(X)$ ensures that the invariant set $\mathcal{F}$ holds for all sequences of non-deterministic choices made at each execution step, while $\texttt{pre}(X)$ ensures that the invariant set $\mathcal{F}$ holds for at least one sequence of non-deterministic choices. Note that $\widetilde{\texttt{pre}}(X) = \texttt{pre}(X)$ for deterministic programs, since $|\texttt{post}(\{\sigma\})| = 1$ for every state $\sigma \in \Sigma$ in this case.

*Abstract semantics.* In order to compute an *under-approximating set of sufficient preconditions*, we require an abstract domain $\mathbb{D}$ with the following backward abstract operators: meet $\sqcap_{\mathbb{D}}^{\texttt{under}}$, backward assignment $\overleftarrow{\texttt{b-assign-under}}_{\mathbb{D}} : \mathit{Var} \times \mathit{Exp} \times \mathbb{D} \times \mathbb{D} \to \mathbb{D}$, backward tests $\overleftarrow{\texttt{b-filter-under}}_{\mathbb{D}} : \mathit{Exp} \times \mathbb{D} \times \mathbb{D} \to \mathbb{D}$, and a lower widening $\underline{\nabla}_{\mathbb{D}}$ [26]. The above abstract operators represent a sound under-approximation of the corresponding concrete operators. We let $\texttt{gfp}^{\#\texttt{under}}$ denote an abstract pre-fixpoint operator, derived using lower widening $\underline{\nabla}_{\mathbb{D}}$, that under-approximates the concrete $\texttt{gfp}$.

We design two backward sufficient condition abstract interpreters that propagate backwards the invariants ensuring that the final assertion is satisfied $d_{\texttt{final}}^{sat}$ and violated $d_{\texttt{final}}^{viol}$, respectively. They are based on a family of backward transfer functions $\overleftarrow{\delta}_{l,l'}^{\texttt{under}} : \mathbb{D} \times \mathbb{D} \to \mathbb{D}$, which for some statements are defined as:

- assignment $l_0 : \texttt{x:=}e; l_1$ **:** $\overleftarrow{\delta}_{l_0,l_1}^{\texttt{under}}(d, d') = \overleftarrow{\texttt{b-assign-under}}_{\mathbb{D}}(\texttt{x}, e, d, d')$
- if statement $l_0 : \textsf{if}\ (e)\ \textsf{then}\ \{l_0^t : s;\ l_1^t\}\ \textsf{else}\ \{l_0^f : s';\ l_1^f\}; l_1$ **:** $\overleftarrow{\delta}_{l_0,l_0^t}(d, d') = \overleftarrow{\texttt{b-filter-under}}_{\mathbb{D}}(e, d, d')$, $\overleftarrow{\delta}_{l_0,l_0^f}(d, d') = \overleftarrow{\texttt{b-filter-under}}_{\mathbb{D}}(\neg e, d, d')$, and $\overleftarrow{\delta}_{l_1^t,l_1}(d, d') = d \sqcap d'$, $\overleftarrow{\delta}_{l_1^f,l_1}(d, d') = d \sqcap d'$
- $l_0 : \texttt{while}\ (e)\ \texttt{do}\ \{l_0^t : s;\ l_1^t\}; l_1$ **:** $\overleftarrow{\delta}_{l_0,l_0^t}(d, d') = \overleftarrow{\texttt{b-filter-under}}_{\mathbb{D}}(e, d, d')$, $\overleftarrow{\delta}_{l_0,l_1}(d, d') = \overleftarrow{\texttt{b-filter-under}}_{\mathbb{D}}(\neg e, d, d')$, and $\overleftarrow{\delta}_{l_1^t,l_0}(d, d') = d \sqcap d'$.

The soundness of $\{\overleftarrow{\delta}_{l,l'}^{\texttt{under}} \mid l, l' \in \mathbb{L}\}$ is written as: $\forall d, d' \in \mathbb{D}, \forall \rho \in \gamma_{\mathbb{D}}(d), \rho' \in \gamma_{\mathbb{D}}(d'), \rho \in \gamma_{\mathbb{D}}(\overleftarrow{\delta}_{l,l'}(d, d')) \implies (l, \rho) \longrightarrow (l', \rho')$.

The backward sufficient condition interpreters are defined as:

$$\overleftarrow{F}^{\#\texttt{under}} = \lambda(I, F).\lambda(l \in \mathbb{L}).\ \sqcap_{\mathbb{D}}^{\texttt{under}} \{\overleftarrow{\delta}_{l,l'}^{\texttt{under}}(I(l), F(l')) \mid l' \in \mathbb{L}\}$$

such that results of backward analyzers are: $\overleftarrow{C}_{sat}^{\#\texttt{under}} = \texttt{gfp}_{(\overrightarrow{\mathcal{I}}^{\#}, F_{\mathbb{D}}^{sat})}^{\#\texttt{under}} \overleftarrow{F}^{\#\texttt{under}}$ and $\overleftarrow{C}_{viol}^{\#\texttt{under}} = \texttt{gfp}_{(\overrightarrow{\mathcal{I}}^{\#}, F_{\mathbb{D}}^{viol})}^{\#\texttt{under}} \overleftarrow{F}^{\#\texttt{under}}$. The sufficient preconditions that the final assertion is satisfied and violated are $d_{\texttt{input}}^{sat,\texttt{under}} = \overleftarrow{C}_{sat}^{\#\texttt{under}}(l_{\texttt{input}})$ and $d_{\texttt{input}}^{viol,\texttt{under}} = \overleftarrow{C}_{viol}^{\#\texttt{under}}(l_{\texttt{input}})$, respectively. We can now compute the under-approximated sets $\mathbb{E}_{sat}^{\#\texttt{under}}$ and $\mathbb{E}_{viol}^{\#\texttt{under}}$ of input environments $\mathbb{E}_{sat}$ and $\mathbb{E}_{viol}$ that definitely lead to satisfaction and violation of the final assertion as:

$$\mathbb{E}_{sat}^{\#\texttt{under}} = \mathbb{E} \cap \gamma_{\mathbb{D}}(d_{\texttt{input}}^{sat,\texttt{under}}), \quad \mathbb{E}_{viol}^{\#\texttt{under}} = \mathbb{E} \cap \gamma_{\mathbb{D}}(d_{\texttt{input}}^{viol,\texttt{under}})$$

such that $\mathbb{E}_{sat}^{\#\texttt{under}} \subseteq \mathbb{E}_{sat}$ and $\mathbb{E}_{viol}^{\#\texttt{under}} \subseteq \mathbb{E}_{viol}$.

*Computing success and failure probabilities.* As before, we instantiate $\mathbb{D}$ with the polyhedra numeric abstract domain, since all under-approximating sound backward operators for it have been implemented in the APRON library [26]. The sufficient preconditions $d_{\texttt{input}}^{sat,\texttt{under}}$ and $d_{\texttt{input}}^{viol,\texttt{under}}$ are under-approximations of $\mathbb{E}_{sat}$ and $\mathbb{E}_{viol}$ respectively, so $\#(d_{\texttt{input}}^{sat,\texttt{under}}) \leq \#(\mathbb{E}_{sat})$ and $\#(d_{\texttt{input}}^{viol,\texttt{under}}) \leq \#(\mathbb{E}_{viol})$. Moreover, the input environments which are not in $\gamma_{\mathbb{D}}(d_{\texttt{input}}^{sat,\texttt{under}})$, that is they are in $\mathbb{E} \backslash \gamma_{\mathbb{D}}(d_{\texttt{input}}^{sat,\texttt{under}})$, may lead to the violation of the assertion. Therefore, we have $\#(d_{\texttt{input}}^{viol,\texttt{under}}) \leq \#(\mathbb{E}_{viol}) \leq \#(\mathbb{E}) - \#(d_{\texttt{input}}^{sat,\texttt{under}})$. By similar reasoning, we can also establish that: $\#(d_{\texttt{input}}^{sat,\texttt{under}}) \leq \#(\mathbb{E}_{sat}) \leq \#(\mathbb{E}) - \#(d_{\texttt{input}}^{viol,\texttt{under}})$. We calculate the *success* and *failure probability* of a program $P$ as follows:

$$\begin{array}{l} \frac{\#(d_{\texttt{input}}^{sat,\texttt{under}})}{\#(\mathbb{E})} \leq \mathbf{Pr^s}(\mathbf{P}) = \frac{\#(\mathbb{E}_{sat})}{\#(\mathbb{E})} \leq \frac{\#(\mathbb{E}) - \#(d_{\texttt{input}}^{viol,\texttt{under}})}{\#(\mathbb{E})} \\ \frac{\#(d_{\texttt{input}}^{viol,\texttt{under}})}{\#(\mathbb{E})} \leq \mathbf{Pr^f}(\mathbf{P}) = \frac{\#(\mathbb{E}_{viol})}{\#(\mathbb{E})} \leq \frac{\#(\mathbb{E}) - \#(d_{\texttt{input}}^{sat,\texttt{under}})}{\#(\mathbb{E})} \end{array} \quad (2)$$

*Example 3.* Consider the program $P_3$ from the beginning of this section. Using two backward sufficient condition analyses, we obtain $d_{\texttt{input}}^{sat,\texttt{under}} = (0 \leq \texttt{j} \leq 5)$ and $d_{\texttt{input}}^{viol,\texttt{under}} = (\bot_{\mathbb{D}})$, and so $\#(d_{\texttt{input}}^{sat,\texttt{under}}) = 6$ and $\#(d_{\texttt{input}}^{viol,\texttt{under}}) = 0$. Thus, the success and failure probabilities are:

$$(60\%)\frac{6}{10} \leq Pr^s(P_3) \leq 1\,(100\%) \quad \text{and} \quad (0\%)\,0 \leq Pr^f(P_1) \leq \frac{4}{10}(40\%) \qquad \square$$

## 6 Implementation

We now describe the implementation and evaluation of the ideas presented so far. The evaluation aims to show the following objectives:

**O1:** The probabilistic analysis can be used to analyze the behaviour of various interesting programs;
**O2:** The probabilistic analysis gives exact results (with no precision loss) in many cases, especially for deterministic programs;
**O3:** The performance time of probabilistic analysis is largely insensitive to domain sizes of input variables;
**O4:** We can find practical application scenarios of using our probabilistic analysis to efficiently analyze C programs.

*Implementation.* We have implemented a prototype probabilistic static analyzer that accepts programs written in a subset of C. It does not support `struct` and `union` types, and provides only a limited support of arrays and pointers. The only basic data types considered are integers. As output, the tool reports the upper and lower bounds of probabilities that the target assertion is satisfied or violated. The prototype tool is written in OCaml. As the abstract analysis domain $\mathbb{D}$ for encoding program properties, we use the polyhedra numeric abstract domain [10]. All abstract operators and sound transfer functions for the polyhedra domain are provided by the APRON library [21,26]. The tool performs one forward reachability analysis and two backward necessary/sufficient condition analyses (one for satisfaction and one for violation of the assertion). The tool calls a model counter, LattE [1], to determine the number of solutions to discovered preconditions for satisfaction or violation of the assertion. Note that if an input state satisfies the discovered precondition for satisfaction (resp., violation) of the assertion, then all program executions branching from that state will satisfy (resp., violate) the given assertion. The analysis proceeds by structural induction on the program syntax, iterating `while`-s until a fixed point is reached. They compute the unique solution which to every program point assigns an element from the abstract domain $\mathbb{D}$.

*Experimental setup and benchmarks.* All experiments are executed on a 64-bit Intel®Core$^{TM}$ i5 CPU, Lubuntu VM, with 8 GB memory. The reported times represent the average runtime of five independent executions. We report Time$_{an}$ to perform all static analyses tasks (one forward plus two backward static analyses), Time$_{pr}$ to compute the needed probability bounds (call to LattE plus additional calculations), and Time to complete the overall probabilistic analysis task. The implementation, benchmarks, and all results obtained are available from: https://aleksdimovski.github.io/probab-analysis.html (or, https://github.com/aleksdimovski/probab_analyzer).

For our experiment, we use a dozen of C programs taken from several folders (categories) of the 8th International Competition on Software Verification (SV-COMP 2019) [6], as well as from the abstract interpretation community [26,30]. The folders from SV-COMP 2019 we consider are: `loops`, `loop-lit`, `termination-crafted` (which is denoted `ter-crafted` for short), as well as `termination-restricted-15` (which is denoted `ter-restricted` for short). We have selected some numeric programs with integers that our tool can handle. We have manually added input sections, and in some of the programs we have also defined target assertions. Then, we have analyzed those programs using our prototype static analyzer. Table 1 summarizes relevant characteristics for each benchmark: the folder (source) where it is taken from, the number of lines of code (LOC), and the number of integer variables (#var). There are two classes of benchmarks in Table 1 separated by a double horizontal line. The first (upper) class of benchmarks consists of deterministic programs for which backward necessary condition analysis is performed, while the second (lower) class of benchmarks are non-deterministic programs for which backward sufficient condition analysis is performed.

*Performances* Table 1 shows the performance of our technique on a set of small and compelling examples (addresses Objective (**O1**)). We can note that for most of our deterministic benchmarks, the technique gives exact results without any approximation (which are marked with ✓ in the Exact column of Table 1). This means that the lower and upper bounds for success and failure probabilities

[6] https://sv-comp.sosy-lab.org/2019/

coincide. This is due to the fact that we use the expressive and very precise polyhedra abstract domain (addresses Objective (**O2**)). For the remaining cases, the technique gives approximate results (which are marked with ≈ in the Exact column of Table 1), since the abstraction was too coarse to calculate exact results. We can also see that the time for static analysis $\text{Time}_{an}$ dominates in the overall probabilistic analysis time Time, whereas the probability computation time $\text{Time}_{pr}$ is a smaller fraction of the total time. The small probability computation times indicate that preconditions obtained from our analyses are relatively simple, and so LattE can handle them very efficiently. We have also experimented with different domain sizes $n$ of input variables (for $n = 10$ and $n = 1000$). Thus, $n$ denotes the number of possible values per input variable. We observe that we obtain similar time performance results for $n = 10$ and $n = 1000$, which means that the performance is not affected by the fact that inputs come from a bigger pool of possible values. This is mostly due to the fact that LattE and APRON are largely insensitive to those values in terms of time (addresses Objective (**O3**)). In general, the obtained probability bounds provide non-trivial information about the behaviour of these programs and are quite hard to estimate by hand even if the programs in question are small.

*Application scenarios.* Consider the following program (called `Waldkirch.c` from `termination-crafted` folder of SV-COMP 2019):

```
① : int x:=[−5,4]; l_input :
② : while (x ≥ 0) {
③ :      x:=x-1; }
l_final : assert (x ≥ −1);
```

We want to prove this assertion, since, for example, later on in the program there are references to an array using the index `x+1` (e.g. `a[x+1]:=0`). In this way, we want to verify that there are no array-out-of-bounds references. The tool will find that the necessary precondition for assertion satisfaction is: $-1 \leq \mathtt{x} \leq 4$, thus computing the success probability of 60%. The found necessary precondition for assertion violation is: $-5 \leq \mathtt{x} \leq -2$, so the failure probability is 40% (addresses Objective (**O4**)).

*Approximate results* We now give an example where we obtain a precision loss in practice due to the approximation inherent in abstract analyses. Consider the following program (taken from [30]):

```
① : int x:=[0,9], y:=[0,9]; l_input :
② : int s:=x-y;
③ : if (s ≥ 2) y:=y+2;
l_final : assert (y > 3);
```

The forward analysis will infer that the program can both satisfy and violate the assertion. The backward necessary condition analysis for assertion satisfaction will discover the constraint: $\mathtt{x} + 2\mathtt{y} \geq 8 \wedge 0 \leq \mathtt{x} \leq 9 \wedge 2 \leq \mathtt{y} \leq 9$, thus we

Table 1: Experimental evaluation for probabilistic static analyses of C programs. This table contains the following columns: (1) *benchmark* - the name of the analyzed program; (2) *source* - the source (folder) where the benchmark is taken from; (3) *LOC* - the number of lines of code; (4) *#var* - the number of integer variables; (5) $\text{TIME}^{10}_{an}$ - the static analysis time in seconds for input domains of size 10; (6) $\text{TIME}^{10}_{pr}$ - the probability computation time in seconds for input domains of size 10; (7-8) $\text{TIME}^{10}$ and $\text{TIME}^{1000}$ - the overall times in seconds required to completely analyze a benchmark which has input domain of size 10 and size 1000, respectively; (9) *Exact* - the preciseness of the reported result (✓ - result is exact, ≈ - result is approximate). Benchmarks above the double horizontal line are deterministic programs, while those below are non-deterministic programs.

| Bench. | source | LOC | #var | $\text{TIME}^{10}_{an}$ | $\text{TIME}^{10}_{pr}$ | $\text{TIME}^{10}$ | $\text{TIME}^{1000}$ | EXACT |
|---|---|---|---|---|---|---|---|---|
| `count_up_down*.c` | `loops` | 20 | 3 | 0.043 | 0.001 | 0.004 | 0.049 | ✓ |
| `hhk2008.c` | `loop-lit` | 20 | 4 | 0.103 | 0.001 | 0.104 | 0.113 | ✓ |
| `gsv2008.c` | `loop-lit` | 20 | 2 | 0.027 | 0.001 | 0.028 | 0.030 | ✓ |
| `Log.c` | `ter-restricted` | 30 | 4 | 0.194 | 0.001 | 0.195 | 0.197 | ≈ |
| `Mono3-1.c` | `loops-crafted-1` | 15 | 2 | 0.044 | 0.001 | 0.045 | 0.046 | ≈ |
| `Waldkirch.c` | `ter-crafted` | 20 | 1 | 0.010 | 0.001 | 0.011 | 0.012 | ✓ |
| `bwd-loop1a.c` | [26] | 15 | 1 | 0.008 | 0.001 | 0.009 | 0.010 | ✓ |
| `bwd-loop2.c` | [26] | 15 | 2 | 0.020 | 0.002 | 0.022 | 0.022 | ✓ |
| `example1a.c` | [30] | 10 | 1 | 0.008 | 0.001 | 0.009 | 0.008 | ✓ |
| `example7a.c` | [30] | 15 | 2 | 0.023 | 0.001 | 0.024 | 0.026 | ✓ |
| `for-bounded*.c` | `loops` | 30 | 4 | 0.049 | 0.002 | 0.051 | 0.053 | ≈ |
| `bwd-loop7.c` | [26] | 15 | 2 | 0.027 | 0.001 | 0.029 | 0.030 | ≈ |
| `bwd-loop10.c` | [26] | 20 | 2 | 0.046 | 0.001 | 0.047 | 0.048 | ≈ |
| `example7b.c` | [30] | 15 | 2 | 0.039 | 0.001 | 0.040 | 0.048 | ≈ |

find that the upper bound probability for assertion satisfaction is 74%. The backward necessary condition analysis for assertion violation will discover the constraint: $\mathtt{x} + 5\mathtt{y} \leq 23 \wedge 0 \leq \mathtt{x} \leq 9 \wedge 0 \leq \mathtt{y} \leq 3$, thus we find that the upper bound probability for assertion violation is 32%. In this way, we conclude that the success probability is between 68% and 74%, while the failure probability is between 26% and 32%. On the other hand, we can calculate by hand that the success probability is exactly 71%, while the failure probability is exactly 29%.

## 7 Related work

Probabilistic analysis of imperative programs based on symbolic execution has been introduced before [18,17,3,29]. They calculate path probabilities by counting the number of solutions to a path condition, which represents a constraint on inputs. The analyses in [18,17] address programs with integer domains and linear constraints, whereas the analyses in [3] address programs with linear and complex floating-point computations. While the previous analyses are restricted to discrete, uniform random variables that take on a finite set of values, the probabilistic analysis in [29] can also handle non-uniform distributions over the reals and integers using a branch-and-bound technique over polyhedra. However, in presence of loops all above analyses based on symbolic execution lose precision, since they cannot enumerate all program paths. The solution is to consider bounded exploration of loops and only a finite number of feasible program paths. Thus, they also define a measure of *confidence* on the obtained probabilistic estimations in order to take into account the contribution from the unexplored feasible paths. For example, if we set the exploration bound of the loop of program $P_2$ in Fig. 2 to any number less than 100, both success and failure probabilities will be 0% and the confidence will be also 0. This is due to the fact that the `while` loop in Fig. 2 has to be unrolled at least 100 times in order to obtain a feasible path on which it can be decided whether the assertion at point $l_{\texttt{final}}$ is satisfied or violated. In this work, instead of symbolic execution we use abstract interpretation to analyze programs and infer preconditions for success and failure. Thus, our approach for computing program reliability represents one of the pioneering works that provides a complete and fast treatment of `while` loops. In particular, the strength of our approach is being an abstract interpretation of a complete semantics for computing program reliability. This is stronger than fixing a priori an incomplete reasoning approach that can miss some feasible program paths (executions). The work [13] performs a probabilistic analysis of open programs using symbolic game semantics [12] and model counting. It uses game semantics to model open programs with undefined identifiers (e.g. calls to library functions), such that the model takes into account all possible contexts in which those programs can be placed. In the presence of loops and undefined functions, bounded exploration in the model is also used to obtain a feasible analysis. Probabilistic model checking [2] is yet another approach to perform probabilistic analysis on a high-level design of software. However, such high-level models are difficult to maintain and may abstract important details that impact the chance of property satisfaction. So the goal is to do probabilistic analysis directly on source code as here, not on high-level models.

Backward precondition analyses by abstract interpretation have also been used in practice for a long time [4,9,26,28]. Sufficient preconditions have been first introduced by Bourdoncle [4] in his work on abstract debugging of deterministic programs. He uses a combination of forward-backward analyses to find preconditions for invariant and intermittent assertions to always hold. Cousot et. al. [9] propose a method for automatically inferring contract preconditions for intermittent assertions. The preconditions extracted by their method are necessary preconditions, i.e. they do not exclude unsafe executions. Mine [26] presents a method for automatically inferring sufficient preconditions of non-deterministic programs by using a polyhedral backward analysis. The under-approximating sound abstract operators for this backward analysis are implemented as part of the APRON library. Rival [28] uses forward-backward analysis to inspect more closely reported alarms by ASTREE, which are then classified as true errors (bugs) or false alarms. Urban and Mine [30] use forward-backward analysis for the automatic inference of sufficient preconditions for program termination. The elements of the analysis domain are decision trees, where decision nodes are labeled with linear numerical constraints and leaf nodes are affine ranking functions for proving program termination. Forward-backward analysis schemes have been used in [20] for the inference of safety properties of declarative synchronous programs. In this work, for the first time we employ forward-backward precondition analysis for estimating program reliability.

Static analysis of probabilistic programs by abstract interpretation has also been a topic of research [27,11]. Monniaux [27] proposes a probabilistic analysis that annotates abstract domains with upper bounds on the probability measure associated with abstract objects. However, the measure bound is associated with the entire abstract object, without tracking how it is distributed amongst the individual states present in the concretization. This restriction makes the analysis quite conservative. Cousot and Monerau [11] provide a general framework that encompasses a variety of probabilistic interpretation schemes. However, no concrete implementation of the above probabilistic abstract interpretations is provided yet. A backward abstract interpretation for probabilistic programs [23] uses expectations that are real-valued functions of the program state and quantitative loop invariants. The automatic inference of such quantitative loop invariants was proposed in the recent work of Katoen et al [22].

## 8 Conclusion

We have presented a new static, abstract interpretation-based approach for computing program reliability, which allows to calculate upper and lower bounds of probabilities that a given assertion is satisfied or violated. We construct a combination of forward-backward abstract analyses, in order to find an approximation of a set of input states which lead to definite satisfaction (resp., violation) of the given assertion. Our approach to calculating program reliability is semantics-based and approximate in a provably sound way. Still, it often yields very precise results, especially for deterministic programs.

We currently support only uniform distribution of input values within their finite discrete domains. In future, we plan to model imprecision in the input by different non-uniform distributions, such as Binomial, Poisson, etc [29]. The current implementation of LattE is limited in handling non-uniform distributions, so we will explore the use of statistical sampling techniques in those cases. Our focus here is on estimating probability for safety properties. We also plan to consider liveness properties (such as termination) and expectation queries [30]. An interesting direction for future work would also be to consider general probabilistic programs [19], as well as program families implemented with `#ifdef`-s from the C-preprocessor where we can use lifted static analyses to efficiently analyze all variants of the family simultaneously at once [14,24,15,16].
